# Developing a Fluorescent Hybrid Nanobiosensor Based on Quantum Dots and Azoreductase Enzyme for Methyl Red Monitoring

**DOI:** 10.29252/ibj.25.1.8

**Published:** 2020-09-12

**Authors:** Fahimeh Hajipour, Sedigheh Asad, Mohammad Ali Amoozegar, Ali Asghar Javidparvar, Jialun Tang, Haizheng Zhong, Khosro Khajeh

**Affiliations:** 1 Department of Biochemistry, Faculty of Biological Science, Tarbiat Modares University, Tehran, Iran;; 2 Department of Biotechnology, College of Science, University of Tehran, Tehran, Iran;; 3 Extremophiles Laboratory, Department of Microbiology, Faculty of Biology, College of Sciences, University of Tehran, Tehran, Iran;; 4 School of Metallurgy and Materials Engineering, College of Engineering, University of Tehran, Tehran, Iran;; 5 Beijing Key Laboratory of Nanophotonics and Ultrafine Optoelectronic Systems, Schoolof Materials Science and Engineering, Beijing Institute of Technology, Beijing, China

**Keywords:** Azoreductase, Methyl red, Quantum dots

## Abstract

**Background:**

Azo dyes are the most widely used synthetic colorants in the textile, food, pharmaceutical, cosmetic, and other industries, accounting for nearly 70% of all dyestuffs consumed. Recently, much research attention has been paid to efficient monitoring of these hazardous chemicals and their related metabolites because of their potentially harmful effect on environmental issues. In contrast to the complex and expensive instrumental procedures, the detection system based on the QDs with the superior optochemical properties provides a new era in the pollution sensing and prevention.

**Methods:**

We have developed a QD-enzyme hybrid system to probe MR in aqueous solutions using a fluorescence quenching procedure.

**Results:**

The azoreductase enzyme catalyzed the reduction of azo group in MR, which can efficiently decrease the FRET between the QDs and MR molecules. The correlation between the QDs photoluminescence recovery and MR enzymatic decolorization at the neutral phosphate buffer permitted the creation of a fluorescence quenching-based sensor. The synthesized biosensor can be used for the accurate detection of MR in a linear calibration over MR concentrations of 5-84 μM, with the LOD of 0.5 μM in response time of three minutes.

**Conclusion:**

Our findings revealed that this fluorometric sensor has the potential to be successfully applied for monitoring a wide linear range of MR concentration with the relative standard deviation of 4% rather than the other method.

## INTRODUCTION

Azo dyes are the most widely used synthetic colorants in the textile, food, pharmaceutical, cosmetic, and other industries, accounting for nearly 70% of all dyestuffs consumed^[^^1^^-^^4^^]^. More than 15% of the used azo dyes are released into textile effluents through prepping fiber, dyeing, and printing processes^[^^5^^-^^7^^]^. Accordingly, industrial effluents often contain remaining dye components, which may lead to water contamination and become a threat to the public health^[^^8^^]^. Although the EU criteria for the classification of dangerous substances have defined that the acute toxicity of azo dyes is rather low^[^^9^^]^, after releasing into the aquatic environment, these substances may cleave to potentially carcinogenic amines that may have a harmful impact on the ecosystem and human health^[^^8^^,^^10^^]^. 

Nowadays, with great global progress and rapid increase in demand for chemicals, the maintenance of human health and wellbeing remains a major concern and one of the most important technological objectives. To achieve this goal, the development of reliable detection methods for accurate determination of pollutions in industrial samples is an urgent need to facilitate the prevention of disease. Various instrumental techniques have been utilized for textile wastewater detection and purification, including activated carbon adsorption^[^^11^^]^, instrumental coagulation-flocculation^[^^12^^]^, advanced oxidation processes^[^^13^^]^, and photocatalytic decomposition^[^^14^^]^, as well as chromatography procedures such as TLC^[^^15^^]^, GC/MS^[^^16^^]^, and HPLC^[^^17^^,^^18^^]^. Despite the extensive use of the above-mentioned routine processes in the wastewaters, these techniques have a number of limitations, including laboratory dependent, high cost, low efficiency, complex operational options, high sludge formation, and limited applicability^[^^19^^,^^20^^]^. Therefore, a great interest in exploring and developing biological sensing systems to monitor the concentration of dye substances in aqueous solutions is growing up^[^^21^^,^^22^^]^. 

In the last decade, significant advances in nanobiotechnology have been created using powerful optoelectronic labels, known as QDs, which can be used in sensor and target-specific probe applications^[^^23^^-^^28^^]^. QDs, semiconductor nanoparticles with diameters of 2-10 nanometers, have attracted attention because of their great optical properties compared to traditional organic fluorophores^[^^29^^-^^31^^]^. For instance, QDs exhibit broad absorption with narrow, size-tunable and symmetric *fluorescence* spectra (full width at half maximum ~25–40 nm)^[^^32^^]^, strong resistance to photobleaching^[^^33^^]^, and high *molar absorption coefficients *(~10–100 × that of organic dyes) with significant luminescence quantum yield^[^^34^^]^. 

Many studies have illustrated the ability of numerous Gram-positive and Gram-negative bacteria to decolorize a large variety of azo dyes^[^^35^^-^^38^^]^. *It is generally accepted that the textile wastewater is characterized by *extremely high salinity ranging from 3.5-20%^[^^39^^]^. The biological removal of color from textile wastewater in this salty environment is performed only in the presence of halotolerant and halophilic microorganisms, which are able to grow and thrive under such harsh conditions^[^^40^^,^^41^^]^. Over the past years, some investigations reported the isolation and characterization of bacterial azoreductases from various bacteria^[^^42^^-^^44^^]^*.* However, to date, only two genes encoding azoreductase enzyme from halophilic bacteria have been isolated and identified^[^^45^^,^^46^^]^. As the first study, Eslami and coworkers in 2016 isolated and characterized an efficient halophilic azoreductase enzyme from *Halomonas elongata IBRC-M10216 *(DSM 2581T)^[^^46^^]^. In this study, we focused on the capability of this halophilic azoreductase in the detection of azo dyes. *Halomonas elongate* reduces azo pollutions produced by textile industry via the use of azoreductase enzyme. This enzyme, which were previously been cloned and characterized in our laboratory, not only has the ability to respond to such harsh environment but also exhibits more efficient kinetic parameters in comparison to enzymes isolated from other bacteria^[^^42^^,^^45^^,^^47^^]^. Hence, in the present study for the first time, we have used the *Halomonas* azoreductase to construct a QDs-based sensor, which allows the monitoring of azo dyes. In 2013, Gromova *et al.*^[^^48^^]^ developed an effective complex of semiconductor CdSe/ZnS QDs with the molecules of azo dyes in polymer track membranes. They reported that the azo dyes, as an electron acceptor on the surface of QDs, could strongly quench the emission of QDs due to their spectral overlap. In another work Annas *et al.*^[^^49^^]^ have demonstrated that the complex of CdSe/ZnS QDs possessed photo-induced dissociation properties with the molecule of azo dye under the function of external radiation of various spectral powers and compositions. They found that the energy transfer from the QDs to the azo dye molecule extremely contributes to the dissociation rate of the complexes. 

Considering these studies, the aim of this work was to describe a water-soluble MPA-capped CdSe/ZnS QD-azoreductase enzyme system for monitoring MR, as a model of azo compounds. In other words, combination of catalytic function of azoreductase enzyme and superior optoelectronic properties of QDs brings up an opportunity to design a preliminarily sensitive QD-based biochemical assay for monitoring azo dyes.

## MATERIALS AND METHODS

**Materials**

Isopropyl β-D-1-thiogalactopyranoside, cadmium oxide (99.99%), NaOH, zinc acetate (99.9%, powder), NaCl, selenium (99.9%, powder), sulfur (99.9%, powder), TOP (90%), OLA (90%), K_2_HPO_4_, KH_2_PO_4,_ OA (90%), 1-dodecanethiol (98%), ODE (90%), and MPA (99.8%) were purchased from Sigma Aldrich (St. Louis, MO, USA). Trypton, yeast extract, and glycine were obtained from Scharlau, Spain. Glycerol, SDS, NiSO_4_, MR, imidazole, and EDTA were obtained from Merck (Darmstadt, Germany). His-tag purification resin and NADH were provided by Roche (Germany). All the reagents were of pro-analysis quality and used in the experiments as received.

**Protein expression and purification**

The recombinant azoreductase enzyme utilized in this study has been produced previously^[^^46^^]^. Briefly, azoreducatse enzyme originally isolated from *Halomonas elongata*. The *E. coli BL21 (DE3)* transformants were grown in LB medium containing ampicillin at 37 °C. Subsequently, when the OD at 600 nm reached about 0.5, IPTG was added to a final concentration of 0.1 mM. After harvesting the cells by centrifugation, the supernatant was loaded onto Ni-NTA agarose column, and then bound proteins were eluted with 200 mM of imidazole in the elution buffer.

**Azoreductase activity assay**

MR, a common mono-azo dye, was selected for further studies. The stock solution of MR was prepared by dissolving 0.02 g of MR in 100 mL of 60% ethanol. The enzyme assay mixture, including 0.1 mM of NADH solution (as a cofactor) and varying concentrations of MR (4–40 µM; as substrate) were added step by step to the phosphate buffer of pH 7 to reach a final assay volume of 0.4 mL. The reaction was initiated with 1-min delay from the addition of azoreductase enzyme, and subsequently, the absorption of NADH at 340 nm over 1 min period decreased through the reaction. In the next step, a calibration graph was generated under the optimum experimental conditions according to the well-known Beer-Lambert equation at 340 nm.

**Synthesis of OLA-capped CdSe/ZnS QDs**

The OLA-capped CdSe/ZnS core shell QDs were prepared according to the previous explanations^[^^50^^-^^52^^]^. Briefly, 1.2 mmol of cadmium oxide, 12 mmol of zinc acetate, 18 mL of OA, and 60 mL of ODE were ‍mixed in a 250-mL round flask. The mixture was degassed at 120 °C under vacuum for 20 min. For the creation of a clear CdSe core solution in the next step, the temperature was set at 300 °C under nitrogen flow with stirring. At this temperature, the Se precursor solution containing 1.3 mmol of selenium powder, 13.3 mmol of sulfur powder, and 10 mL of TOP were swiftly loaded into the reaction flask to promote the growth of QDs for 10 min. The mixture of 15 mmol of Zn(OAc), 10 mL of OLA, 15 mL of ODE, and 15 mL of DDT was degassed at 80 °C for 30 min, and then the temperature was raised up to 120 °C in nitrogen atmosphere until the Zn stock solution became transparent. In the following phase, the shell precursor was injected dropwise (~2 mL/min) into the hot flask for a period of times to increase the efficiency of QDs for energy transfer. After purification and precipitation by adding excess methyl alcohol, anhydrous, and acetone, the resulting CdSe/ZnS QDs were dispersed in a nonpolar solvent (methyl benzene, chloroform) or dried to powder. The preparation of water-soluble CdSe/ZnS QDs for biosensing applications was based on a previous study^[^^52^^]^ in which the ligand exchange method was applied to replace OLA at the surface of nanoparticles with MPA^[^^52^^]^. For this purpose, 2 mL of MPA was added to the dispersion of 60 mg of OLA-capped QDs and 5 mL of N,N-Dimethylformamide in a 100-mL round flask. The degassed reaction mixture was incubated at 130 °C until a clear solution was formed with the protection of nitrogen. After precipitating MPA-capped QDs by adding 2-propanol, the pellet was re-dissolved in alkaline buffer solution (pH ∼12) for storage.

**Instrumentation and apparatus**

During the preparation of recombinant azoreductase and characterization of MR and CdSe/ZnS QDs, ultraviolet-visible and fluorescence spectroscopic studies were carried out using Perkin Elmer Lambda 25 UV/VIS spectrophotometer and Perkin Elmer Luminescence Spectrometer LS 55, respectively. FTIR spectroscopy (Perkin Elmer) was conducted on the KBr pellets of the synthesized QDs in the region of 400–4000 cm^-1^. The TEM images of the purified CdSe/ZnS QDs were captured on a JEOL JEM-2100F microscope operating at 200 kV. The XRD measurements of CdSe/ZnS QDs were performed on a Rigaku D/Max-2500 X-ray diffractometer.

**Fluorescence experiments**

The basic sample for fluorescence analysis was created by adding 200 µL of 5 mM of NADH stock solution to 20 µL of QDs in 50 mM of phosphate buffer solution (pH 7). In order to create the fluorescence quenching-based detection procedure for azo dyes, QDs with PL peak at 520 nm, as a donor, was coupled with MR, as an acceptor, in energy transfer processes. In all the experiments, the fluorescence intensities were measured under the excitation and emission wavelengths of 365 and 520 nm, respectively. To explore the effect of different amounts of MR on the fluorescence intensity of QD, 

**Fig. 1:**
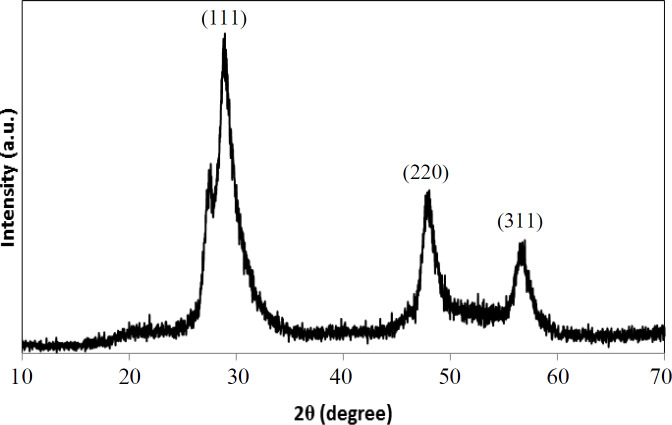
XRD patterns of CdSe/ZnS core-shell QDs

we diluted 100 µL of QD mother solution to 3 mL with phosphate buffer solution of pH 7, in the presence of MR over the range of 10 to 150 µM. Next, the fluorescent intensity of each mixture was measured spectrophotometrically at 520 nm after incubating for 3 min. In the next step, for studying the impact of azoreductase enzyme on each sample, 24 µg/ml of the enzyme was transferred to tubes. All the experiments in this study were repeated at least three times.

## RESULTS AND DISCUSSION

 Due to the stability and xenobiotic nature, azo dyes are not completely degraded by conventional wastewater treatment procedures. Therefore, it is important to detect these pollutants in industrial effluents before their discharge into the environment. This work aimed to develop a fluorescence quenching-based method, which has received growing interest in the QD-based sensing field, for monitoring MR before discharging into the surface water. The detection was carried out by FRET modulation between QDs and MR component^[^^53^^-^^57^^]^. Because the synthesized OLA-capped CdSe/ZnS QDs were initially soluble in organic solvents, it was necessary to render them water soluble for biological applications. The aqueous phase transfer of samples was achieved by replacing the OLA using MPA.

MPA has two functional groups, carboxyl and thiol. The thiolic end displayed a strong electron affinity to the zinc in the outer ZnS shell, and thus the initial surface ligand could be replaced with MPA. Carboxyl group existing in the molecular structure also provided the water solubility to QDs because of its strong participation in hydrogen bond formation^[^^51^^,^^52^^,^^58^^-^^60^^]^. The successful phase transfer of these nanoparticles after ligand exchange could be directly illustrated by the transparency of the resulting solutions. The structural properties of the CdSe/ZnS semiconductor QDs were investigated by XRD test. According to the XRD pattern (Fig. 1), the sample had three main peaks at 2θ = 28.8, 48.2, and 56.9 degrees, which could be indexed as (111), (220), and (311) crystal plates, respectively. Based on the corresponding reference (#65-0309), the peaks of CdSe/ZnS QDs were shifted to higher 2θ values. This shift may be attributed to the compressive strain of CdSe core by ZnS shell. Therefore, these peaks confirmed the successful fabrication of CdSe/ZnS QDs^[^^61^^,^^62^^]^. 

**Fig. 2:**
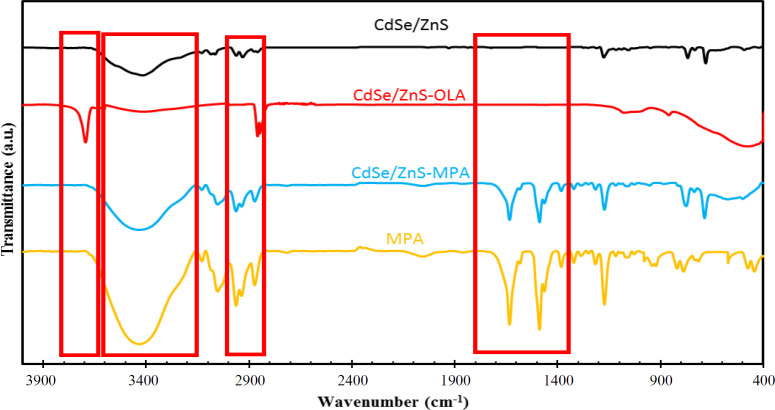
FTIR spectra of CdSe/ZnS, CdSe/ZnS-OLA, and CdSe/ZnS-MPA QDs

The crystalline size of the synthesized QDs calculated by Debye–Scherer equation was found to be ~3.52 nm. The surface treatment of the synthesized QDs can be understood from the FTIR spectra (Fig. 2). As it can be seen in this Figure, the FTIR spectra of CdSe/ZnS-OLA showed a sharp peak at 3744 cm^-1^, which is related to the stretching vibration of N-H bonds, indicating that the NH_2_-containing ligand was adsorbed on the synthesized QD surface^[^^63^^]^. The broad absorption peak at about 3200-3600 cm^-1^ can be assigned to the –OH stretching vibration of physically adsorbed water molecules in CdSe/ZnS and CdSe/ZnS-MPA samples^[^^64^^]^. The significant reduction of the peak in CdSe/ZnS-OLA sample indicates that the sample has more hydrophobic property than the two other samples. Both C–H symmetric and asymmetric stretching vibrations show a characteristic peak at about 3000 cm^-1[^^65^^]^. The presence of C=O and C–O vibration peaks at 1635 and 1488 cm^-1^, respectively, resulted that the MPA molecules successfully replaced with OLA components on the QD surface^[^^66^^]^.

**Fig. 3:**
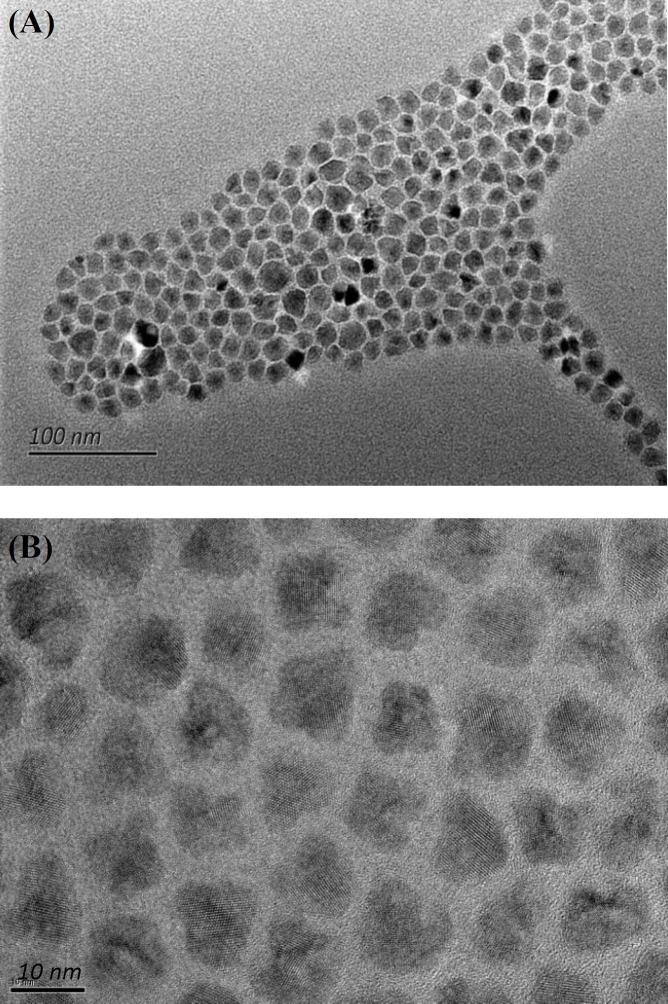
TEM image of the synthesized QDs with the scale bars of 100 nm (A) and 10 nm (B).

**Fig. 4:**
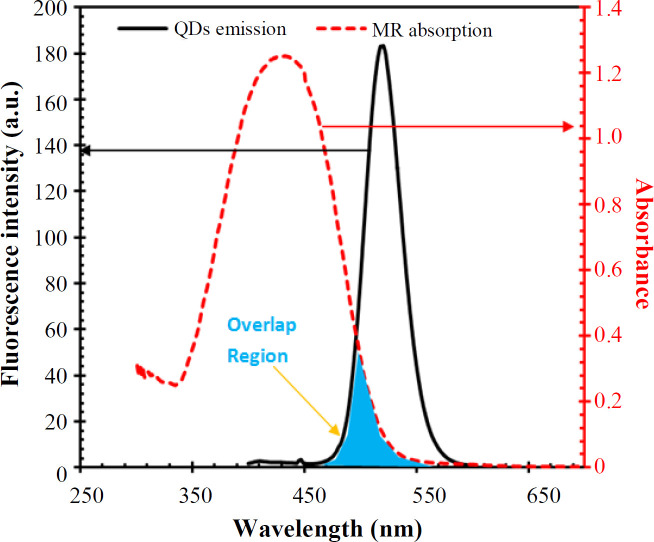
Spectral overlap between QDs emission (20 μl of QD stock solution) and MR (50 μM) absorbance resulted in the fluorescence energy transfer and quenching

The TEM images of the synthesized QDs are shown in Figure 3, which depicts that the nanoparticles are spherical in shape and about 10.5 nm in diameter. Also, the results revealed that the well-dispersed nanoparticles possessed a uniform particle size. The MR absorption bond and emission spectrum of QDs are shown in Figure 4. It can be observed from this Figure that the absorption spectrum of MR exhibits a significant absorption band between 450 and 550 nm coincident with the emission spectrum of QDs. In other words, the emission spectrum of QDs, as donor molecules, overlapped with the absorption band of MR, as an acceptor. Therefore, it is expected that this spectral overlap permits a non-radiative energy transfer between the donor QDs and acceptor MR molecules. When energy is transferred from the excited electronic state QDs to the nearby acceptor chromophore, the FRET unveils itself through decreasing or quenching of the donor fluorescence^[^^67^^]^.

**Fig. 5:**
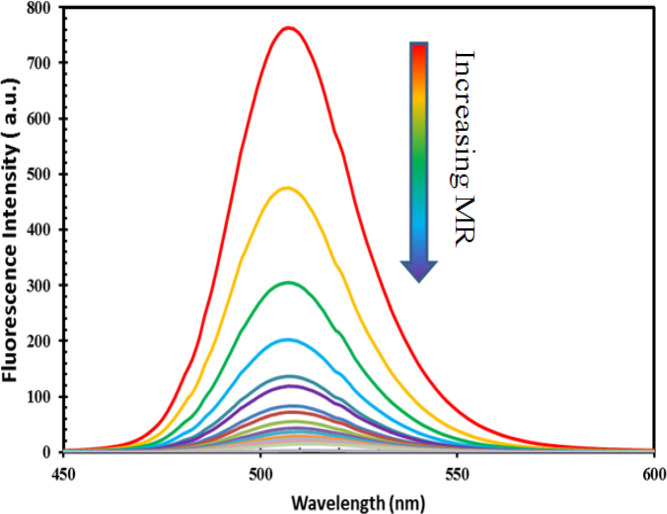
Fluorescence quenching of QDs (80 μl of QD stock solution) in the presence of various concentrations of MR

 To investigate the modification of PL intensity of QDs by the addition of MR (as quencher), the MR was added to QDs in the cuvette, and then the fluorescence of QDs was checked by using a fluorescence spectrometer. Figure 5 shows how the presence of MR in the reaction cell is able to quench the emission of QDs. As observed, the PL intensity of QDs up to 95% decreased gradually as the amount of MR increased in *the cuvette. *

**Fig. 6:**
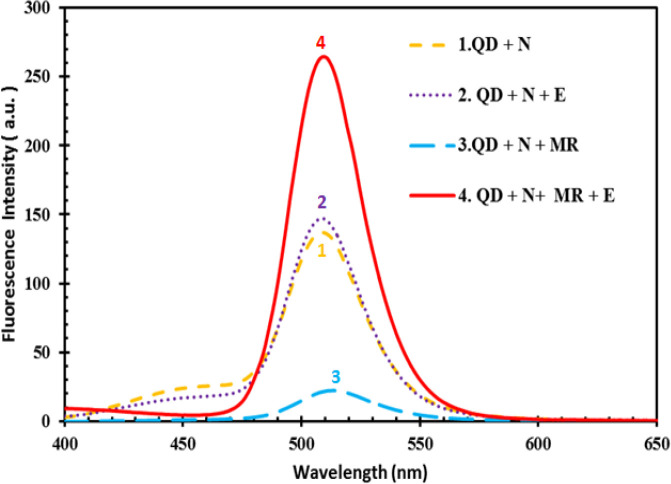
Quenching effect of 20 µM of MR (methyl red) on the QD (quantum dot) emission (20 μl of QD stock solution) in the presence of NADH (N; 0.3 mM) and enzyme (E; 24 µg/ml).

To study the influence of enzyme on the modulation of FRET efficiency between QDs and MR in separate experiments, the PL intensity of QD-NADH mixture, as the reference sample, was studied in different combinations of MR, enzyme, and MR-enzyme mixture (Fig. 6). For this purpose, the fluorescence intensity of QDs before and after the enzymatic cleavage of the quencher was compared. The results depicted in Figure 6 demonstrated that no quenching effect of enzyme on QDs emission was detected in the presence of enzyme alone, whereas MR significantly suppressed the PL intensity of QDs.

**Fig. 7:**
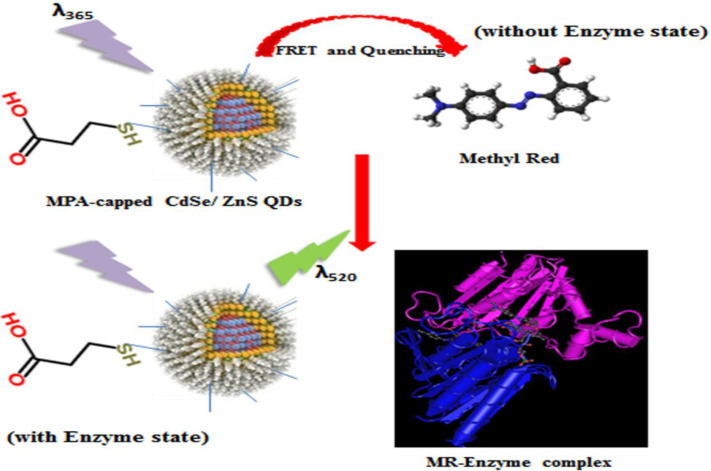
Schematic illustration of MR monitoring by the QD-enzyme hybrid system^[68]^

By incorporation of the enzyme and MR into the reaction mixture, upon the formation of substrate-enzyme complexes as well as reduction of the azo group in MR, the PL intensity of QDs increased significantly from less than 50 to 260. These experiments provide enough evidence in support of this fact that the quenching could be only due to the interaction between the CdSe/ZnS nanoparticles in the excited state as the donor and MR molecules as the acceptor. In this way, the correlation between MR concentration and QDs emission enhancement provides a basis for the creation of a QDs-based sensor for accurate detection of MR. This nanoparticle-based MR sensor comprises a photostable semiconductor QDs and azoreductase enzyme. A schematic illustration of MR monitoring by the QD-enzyme hybrid system is depicted in Figure 7. As shown in the Figure, the QD donors and MR acceptors constitute an efficient FRET pair wherein the MR can strongly quench the emission of QDs. Since the fluorescence of QDs is very sensitive to their surface conditions, in the presence of azoreductase enzyme in reaction, the enzymatic reduction of MR occurred at the surface of QDs, leading to a decrease in the FRET efficiency between the CdSe/ZnS QDs and MR molecules. Accordingly, we can see a correlation between the azo dye removal rate and PL intensity enhancement of QDs, which is very useful for monitoring MR.

**Fig. 8:**
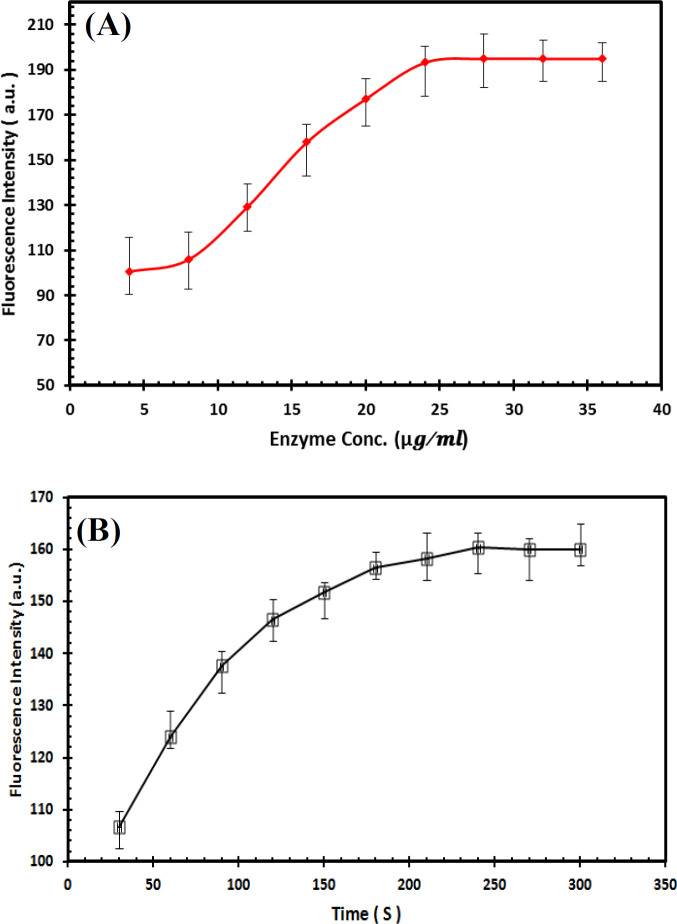
Effect of enzyme concentration and time on the MR-QD FRET ratio. **(A)** Quenching effect of 80 μM of MR in the presence of various azoreductase enzyme concentrations (4, 8, 12, 16, 20, 24, 28, 32, and 36 µg/ml). **(B)** Quenching kinetic effect of 50 μM of MR at different incubation times of 30 s to 300 s in the presence of 24 µg/ml of enzyme concentration

It is important to ensure that whether the presence of azoreductase enzyme in the reaction mixture has a direct significant effect on the FRET efficiency between MR and QDs. In this regard, a reaction cell containing a reference sample with 80 μM MR in a total volume of 3 mL with phosphate buffer was treated with the successive concentrations of the enzyme (from 4 to 36 µg/ml). In order to explore the effect of increasing azoreductase enzyme concentrations on the azo group reduction rate and QD:MR FRET behavior, we used the high saturating MR and NADH concentrations in the reaction^[^^69^^,^^70^^]^. By increasing the enzyme concentration and then incubation for 3 min, the azo dye removal rate will be enhanced gradually, and then decreasing the energy

acceptors per QD donors leads to the high restoration of QD fluorescence. Quenching effect of 80 μM MR in the presence of various azoreductase enzyme concentrations as well as quenching kinetic effect of 50 μM MR at different incubation times can be seen in Figure 8, which represents that the energy transfer between MR and QDs is very sensitive to the enzyme concentration in the range of 4 to 24 µg/ml. The addition of enzyme more than 24 µg/ml to the reaction cell had no effect on the fluorescence of QD solution, signifying that there is not any more free substrate. This observation suggests that there is a positive correlation between the enzyme concentration and the emission enhancement of QDs. In order to further characterize the effective function of enzyme on the FRET modulation between QDs and MR, we carried out the same quenching study in the presence of enzyme over a period of time. In this context, the QDs PL restoration was examined in the presence of 50 μM of MR and 24 µg/ml of azoreductase enzyme in a total volume of 3 mL at different incubation times ranging from 30 to 300 s. Time course of incubation in Figure 8B shows that by increasing the incubation time, more MR molecules were converted to the aromatic amines, and the plateau level of QD PL restoration was reached after approximately 180-s incubation, meaning all the MR molecules in the reaction medium have to be taking part in the reaction in this time. In other words, from the relationship between the change of PL intensity of QD and incubation time, it could be known that the PL intensity of QD was relatively insensitive to the incubation time longer that 180 s. Based on these results, the 24-µg∕ml concentration of azoreductase enzyme and incubation time of 180 s were chosen as the optimum condition for the subsequent experiments of FRET-based detection method. It is clear from the Figure 8 that the NADH, as cofactor, in this system shows a week quenching effect on the QD emission with low efficiency. As indicated in Figure 8, NADH, as cofactor, in this system can slightly induce the QD fluorescence quenching in comparison with MR. The enzymatic reduction of MR was synchronized with the oxidation of NADH to NAD^+^ and also with the removal of the small quenching effect of NADH. In other words, as expected during the enzymatic reduction, the emission intensity of QD gradually enhanced with increasing oxidation of NADH to NAD^+^. 

**Fig. 9:**
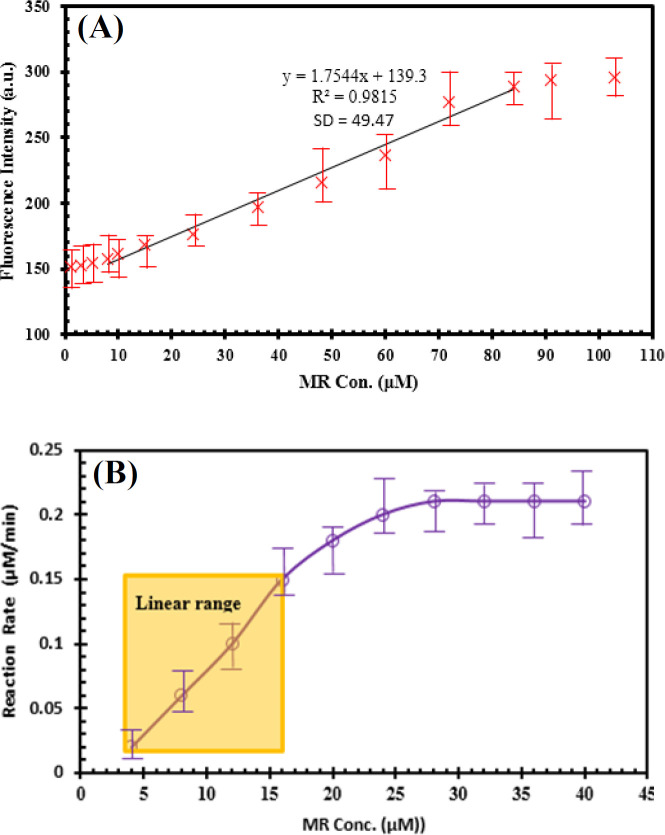
Linear range of detection. (A) Calibration curve for MR monitoring with this nanobiosensor in the linear range of 5 to 84 μM of MR and LOD of 0.5 μM. (B) Kinetic study of the NADH-based reduction of MR with the linear range of 4 to 16 µM of MR and LOD of 1.6 µM

To gain further insight into the sensor, the linear working range and the low detection limit of the nanobiosensor for MR were evaluated as a simple model of azo dyes. To calibrate this sensor, different concentrations of MR were injected into the reference sample to quench the QDs, and azoreductase enzyme was added afterwards to each the quenched sample and allowed to incubate for 3 min. The MR concentration was then plotted against the fluorescence intensity of QDs to generate a calibration plot in three separate experiments (Fig. 9A). 

Using the calibration curve, the linear range of detection for MR (referred to as quencher) was 5 to 84 μM and the lowest detectable concentration of MR without the effect of background signal was 0.5 μM. This high relative LOD in comparison with the other fluorescent sensors is due to the slight interference of NADH in the QD emission intensity through the resonance energy transfer. Owing to this issue, we added NADH to QD solution as a basic sample for the subsequent fluorescent analysis throughout the study. With the incremental enzymatic reduction of MR, the fluorescent intensity of QD was progressively increased along with the oxidation of NADH to NAD^+^ and then removal of quenching effect of NADH. Because the titration of restoration of QD emission after this enzymatic reaction was used to determine MR, a reasonable explanation for the high LOD of the sensor is likely the NADH quenching effect. It should be noted that this study is the first step toward the QD-enzyme hybrid system utilization into azo dyes detection, and the research into solving this drawback is in progress.

**Fig. 10:**
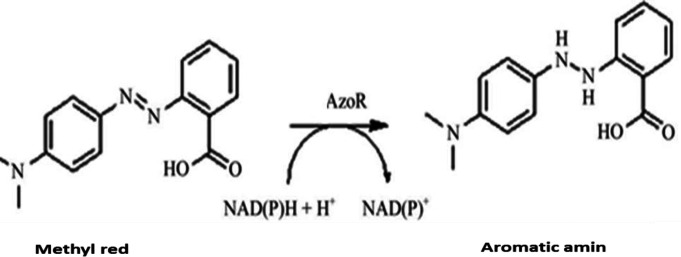
Enzymatic reduction of MR^[75]^.

In our future work, we intend to focus on the optimization of this nanobiosensor together with the other recombinant enzymes such as laccase. The repeatability of the proposed MR nanobiosensor response to 20 µM MR was further examined. The relative standard deviation was 4% for nine consecutive experiments. To evaluate the QD-based sensor, we performed a comparison between this fluorescent sensor and the typical spectroscopic enzyme assay as a color degradation reaction. Some previous studies have reported a number of NAD^+^/NADH-based systems as suitable conventional methods for monitoring NADH^-^associated reactions^[^^71^^-^^73^^]^. In those investigations, the detection processes were on the basis of optical modifications taking place in the medium after the the enzymatic oxidation of NADH. Reduction of the azo groups was carried out using the enzyme-mediated transfer of reducing equivalents generated by the oxidation of coenzymes to azo dyes through a two-step *ping-pong Bi-Bi mechanism*^[^^74^^]^*. *In this study, as shown in Figure 10, during the enzymatic reaction, NADH transferred electrons to the azo dye as the electron acceptor, forming the corresponding oxidized form, NAD^+^. Accordingly, this approach provides simple capabilities for spectrophotometric assay of methyl based on the azoreductase enzymatic activity coupled with the absorption modification of NADH as cofactor in 340 nm. The oxidation of NADH and the amounts of MR consumed during the reaction were monitored by UV absorption spectroscopy.

The data in the calibration curve for NADH oxidation in Figure 9B indicate that the response of the NADH-based method for MR was linear in the range of 4 to 16 µM with the LOD of 1.6 µM. The data from the comparison of QD-based system with azoreductase enzyme assay revealed that the fluorescent approach detected a better correlation between the PL intensity and MR concentration in the upper linear detection range rather than the other method. 

Besides conventional methods, with emerging the QDs into the research fields, some QD-based detecting systems have been reported for various azo dyes. In 2013, Zhou *et al.*^[^^76^^]^ introduced a new luminescence sensing system based on the quenched fluorescence signal of the OA-functionalized Mn-ZnS QDs for the sensitive determination of Sudan dyes in foodstuffs with LODs of 2.1-32.7 ng/ml for different dyes. In a similar study in 2017, Zhang J *et al.*^[^^77^^]^ used the CdTe QDs to present a fluorescence-quenching method for the quantitative analysis of Ponceau 4R as a food azo dye in the linear response of 2.5-25 µg/ml. To date, only one enzyme-based sensor has been reported to detect azo dyes. Mazlan *et al.*^[^^78^^]^ developed a new biosensor for the determination of azo dye tartazine based on the immobilization of laccase enzyme on the functionalized methacrylate-acrylate microspheres. Yin *et al.*^[^^79^^]^ also fabricated a new amperometric sensor based on immobilized CoTe QDs and PAMAM dendrimer onto glassy carbon electrode to detect bisphenol A, as a hazardous material in the environment, with the linear range of 1.3 × 10^-8^ to 9.89 × 10^-6^ M. In addition to sensing and diagnostics applications of QDs, several studies have indicated the medical significance of QDs in various fields such as drug delivery, therapeutics, and imaging^[^^80^^-^^83^^]^. A QD-based procedure was developed by Wang *et al.*^[^^84^^] ^for the detection of ovarian cancer marker CA125 by conjugating the streptavidin-coated QDs to CA125 monoclonal antibodies. In another study, a PEGylated QD core, as a scaffold, was utilized as a delivery vehicle to release the siRNA into specific subcellular targets^[^^85^^]^. In this study, for the first time, using the superior optical properties of QDs and the specificity of enzymatic reaction, we proposed a simple nano-biosensor for MR detection. We intend to focus on this goal with the novel ideas such as utilization of the other more efficient enzyme such as laccase, which can catalyzes the decolorization of azo dyes with no need to NADH^[^^86^^]^. Serious concerns related to cadmium-based toxicity leads to the fabrication of cadmium-free QDs for the environmental safety purposes. To improve the manufacture of nanocrystals in hot-injection process, efforts have been ongoing to use efficient solvents with low toxicity and more stability at high temperature instead of TOP. In order to facilitate the application of this suggested sensor for *in situ* analysis set up, this sensor with different real company effluents is ongoing. Therefore, the creation of analytical techniques such as the present sensor with emphasis on food safety and environmental monitoring fields coordinates well with human wellbeing.

In the present work, we have demonstrated the use of a water-soluble CdSe/ZnS QD azoreductase enzyme nanobiosensor based on FRET for the fluorescent detection of MR that could be extended for different substrates. The results showed that the PL intensity of CdSe/ZnS QDs increased during the gradual enzymatic decolorization of MR, which leads to a significant decrease in the FRET signal between the QDs and MR. Enzymatic reduction of soluble MR produced a concentration-dependent QD PL recovery, due to the elimination of the MR away from the QDs. This good linear correlation between the signal intensity enhancement and the MR concentration in aqueous solution carries new capabilities for optical sensing of MR based on a fluorescence quenching method. 

In summary, this work reports the first example of the utilization of QD enzyme hybrid system as a facile, simple, and cost-effective sensor for the detection of methyl red as a typical azo dye based on the fluorescence quenching.

## References

[1] Saxe JP, Lubenow BL, Chiu PC, Huang CP, Cha DK (2006). Enhanced biodegradation of azo dyes using an integrated elemental iron‐activated sludge system: I Evaluation of system performance. Water environment research.

[2] Hao J, Song F, Huang F, Yang C, Zhang Z, Zheng Yi, Tian X (2007). Production of laccase by a newly isolated deuteromycete fungus pestalotiopsis sp and its decolorization of azo dye. Journal of industrial microbiology and biotechnology.

[3] Brosillon S, Djelal H, Merienne N, Amrane A (2008). Innovative integrated process for the treatment of azo dyes: coupling of photocatalysis and biological treatment. Desalination.

[4] Ledakowicz S, Solecka M, Zylla R (2001). Biodegradation, decolourisation and detoxification of textile wastewater enhanced by advanced oxidation processes. Journal of biotechnology.

[5] Robinson T, McMullan G, Marchant R, Niam P (2001). Remediation of dyes in textile effluent: a critical review on current treatment technologies with a proposed alternative. Bioresource technology.

[6] D'Souza DT, Tiwari R, Sah AK, Raghukumar C (2006). Enhanced production of laccase by a marine fungus during treatment of colored effluents and synthetic dyes. Enzyme and microbial technology.

[7] Puvaneswari N, Muthukrishnan J, Gunasekaran P (2006). Toxicity assessment and microbial degradation of azo dyes. Indian journal of experimental biology.

[8] Konstantinou IK, Albanis TA (2004). TiO2-assisted photocatalytic degradation of azo dyes in aqueous solution: kinetic and mechanistic investigations: a review. Applied catalysis B.

[9] Novotný C, Dias N, Kapanen A, Malachová K, Vándrovcová M, Tävaara M, Lima N (2006). Comparative use of bacterial, algal and protozoan tests to study toxicity of azo-and anthraquinone dyes. Chemosphere.

[10] Fuh MR, Chia KJ (2002). Determination of sulphonated azo dyes in food by ion-pair liquid chromatography with photodiode array and electrospray mass spectrometry detection. Talanta..

[11] Yu Y, Jimmy CY, Chan CY, Che YK, Zhao JC, Ding L, Ge WK, Wong PK (2005). Enhancement of adsorption and photocatalytic activity of TiO2 by using carbon nanotubes for the treatment of azo dye. Applied catalysis B: Environmental.

[12] Lau YY, Wong YS, Teng TT, Morad N, Rafatullah M, Ong SA (2014). Coagulation-flocculation of azo dye Acid Orange 7 with green refined laterite soil. Chemical engineering journal.

[13] Kusvuran E, Gulnaz O, Irmak S, Atanur OM, Yavuz HL, Erbatur O (2004). Comparison of several advanced oxidation processes for the decolorization of reactive red 120 azo dye in aqueous solution. Journal of hazardous materials.

[14] Grzechulska J, Morawski AW (2002). Photocatalytic decomposition of azo-dye acid black 1 in water over modified titanium dioxide. Applied catalysis B: Environmental.

[15] Pielesz A, Świerczek S, Włochowicz A, Baranowska I (1999). Adsorption and partition TLC separation of MAK-type aromatic amines, reduction products of azo dyes. Journal of planar chromatography-modern TLC.

[16] Stylidi M, Kondarides DI, Verykios XE (2003). Pathways of solar light-induced photocatalytic degradation of azo dyes in aqueous TiO2 suspensions. Applied catalysis B: Environmental.

[17] Pielesz A, Baranowska I, Rybak A, Włochowicz A (2002). Detection and determination of aromatic amines as products of reductive splitting from selected azo dyes. Ecotoxicology and environmental safety.

[18] Pielesz A, Baranowska I, Świerczek S, Less AW (1999). Separation of aromatic amines of MAK group, which are the reduction products of azo dyes by partition HPLC chromatography. Chemia analityczna.

[19] Yang HY, He CS, Li L, Zhang J, Shen JY, Mu Y, Yu HQ, Yang HY (2016). Process and kinetics of azo dye decolourization in bioelectrochemical systems: effect of several key factors. Scientific reports.

[20] Punzi M, Anbalagan A, Börner RA, Svensson BM, Jonstrup M, Mattiasson B (2015). Degradation of a textile azo dye using biological treatment followed by photo-Fenton oxidation: evaluation of toxicity and microbial community structure. Chemical engineering journal.

[21] Shah MP (2014). Azo dye reduction by methanogenic granular sludge exposed to oxygen. International journal of environmental bioremediation and biodegradation.

[22] Almeida E, Corso C (2014). Comparative study of toxicity of azo dye Procion Red MX-5B following biosorption and biodegradation treatments with the fungi Aspergillus niger and Aspergillus terreus. Chemosphere.

[23] Luo X, Morrin A, Killard AJ, Smyth M (2006). Application of nanoparticles in electrochemical sensors and biosensors. Electroanalysis.

[24] Jianrong C, Yuqing M, Nongyue H, Xiaohua W, Sijiao L (2004). Nanotechnology and biosensors. Biotechnology advances.

[25] Malik P, Katyal V, Malik V, Asatkar A, Inwati G, Mukherjee TK (2013). Nanobiosensors: concepts and variations. ISRN Nanomaterials.

[26] Iñarritu I, Torres E, Topete A, Campos-Terán J (2017). Immobilization effects on the photocatalytic activity of CdS quantum Dots-Horseradish peroxidase hybrid nanomaterials. Journal of colloid and interface science.

[27] Nie S, Xing Y, Kim GJ, Simons JW (2007). Nanotechnology applications in cancer. Annual review of biomedical engineering.

[28] Smith AM, Duan H, Mohs AM, Nie S (2008). Bioconjugated quantum dots for in vivo molecular and cellular imaging. Advanced drug delivery reviews..

[29] Algar WR, Krull UJ (2008). Quantum dots as donors in fluorescence resonance energy transfer for the bioanalysis of nucleic acids, proteins, and other biological molecules. Analytical and bioanalytical chemistry.

[30] Martín-Palma RJ, Manso M, Torres-Costa V (2009). Optical biosensors based on semiconductor nanostructures. Sensors (Basel).

[31] Medintz IL, Uyeda HT, Goldman ER, Mattoussi H (2005). Quantum dot bioconjugates for imaging, labelling and sensing. Nature materials.

[32] Ibnaouf K, Prasad S, Hamdan A, Alsalhi M, Alswayyan AS, Zaman MB, Masilamani V (2014). Photoluminescence spectra of CdSe/ZnS quantum dots in solution. Spectrochimica acta part A: Molecular and biomolecular spectroscopy.

[33] Hu D, Chen L, Liu K, Xiong J, Kim H (2012). Characteristics of Photobleaching of Quantum Dots CdSe in FBS Solutions. Advances in technology and management.

[34] Vasudevan D, Gaddam RR, Trinchi A, Cole I (2015). Core-shell quantum dots: Properties and applications. Journal of Alloys and Compounds.

[35] Suzuki Y, Yoda T, Ruhul A, Suqiura W (2001). Molecular cloning and characterization of the gene coding for azoreductase from Bacillus sp OY1-2 isolated from soil. Journal of biological chemistry.

[36] Blümel S, Knackmuss HJ, Stolz A (2002). Molecular cloning and characterization of the gene coding for the aerobic azoreductase from xenophilus azovorans KF46F. Applied environmental microbiology.

[37] Blümel S, Stolz A (2003). Cloning and characterization of the gene coding for the aerobic azoreductase from Pigmentiphaga kullae K24. Applied microbiology and biotechnology.

[38] Morrison JM, Wright CM, John GH (2012). Identification, Isolation and characterization of a novel azoreductase from Clostridium perfringens. Anaerobe.

[39] He H, Chen Y, Li X, Cheng Y, Yang C, Zeng G (2017). Influence of salinity on microorganisms in activated sludge processes: A review. International biodeterioration and biodegradation.

[40] Carliel CM, Barclay SJ, Naidoo N, Bukley CA, Mulholland DA, Senior E (1994). Anaerobic decolorisation of reactive dyes in conventional sewage treatment processes. Water SA.

[41] Manu B, Chaudhari S (2003). Decolorization of indigo and azo dyes in semicontinuous reactors with long hydraulic retention time. Process biochemistry.

[42] Macwana SR, Punj S, Cooper J, Schwenk E, John GH (2010). Identification and isolation of an azoreductase from Enterococcus faecium: Oklahoma State University. Current issues in molecular biology.

[43] Misal SA, Lingojwar DP, Shinde RM, Gawai R (2011). Purification and characterization of azoreductase from alkaliphilic strain Bacillus badius. Process biochemistry.

[44] Moutaouakkil A, Zeroual Y, Dzayri FZ, Talbi M, Lee K, Blaghen M (2003). Purification and partial characterization of azoreductase from Enterobacter agglomerans. Archives of biochemistry and biophysics.

[45] Tian F, Guo G, Zhang C, Yang F, Hu ZH, Liu C, Wang SW (2019). Isolation, cloning and characterization of an azoreductase and the effect of salinity on its expression in a halophilic bacterium. International journal of biological macromolecules.

[46] Eslami M, Amoozegar MA, Asad S (2016). Isolation, cloning and characterization of an azoreductase from the halophilic bacterium halomonas elongata. International journal of biological macromolecules.

[47] Punj S, John GH (2009). Purification and identification of an FMN-dependent NAD (P) H azoreductase from Enterococcus faecalis. Current issues in molecular biology..

[48] Gromova YA, Orlova AO, Maslov VG, Fedorov AV, Baranov A (2013). Fluorescence energy transfer in quantum dot/azo dye complexes in polymer track membranes. Nanoscale research letters.

[49] Annas KI, Gromova YA, Orlova AO, Maslov VG, Fedorov AV, Baranov AV (2014). Photoinduced dissociation of complexes of cadmium selenide quantum dots with azo dye molecules. Journal of optical technology.

[50] Chang S, Zhang X, Wang Z, Han D, Tang J, Bai Z, Ahong H (2017). Alcohol-soluble quantum dots: enhanced solution processability and charge injection for electroluminescence devices. Journal of selected topics in quantum electronics.

[51] Zhao C, Bai Z, Liu X, Zhang Y, Zou B, Zhong H (2015). Small GSH-capped CuInS2 quantum dots: MPA-assisted aqueous phase transfer and bioimaging applications. ACS applied materials and interfaces.

[52] Bai Z, Ji W, Han D, Chen L, Chen B, Shen H, Zou B, Zhong H (2016). Hydroxyl-terminated CuInS2 based quantum dots: toward efficient and bright light emitting diodes. Chemistry of materials.

[53] Rosenthal SJ, Chang JC, Kovtun O, McBride JR, Tomlinson ID (2011). Biocompatible quantum dots for biological applications. Chemistry and biology.

[54] Ma Q, Su X (2011). Recent advances and applications in QDs-based sensors. Analyst.

[55] Dibyendu D, Jaba S, Roy AD, Bhattacharjee D, Hussain SA (2014). Development of an ion-sensor using fluorescence resonance energy transfer. Sensors and Actuators B: Chemical.

[56] Oh E, Hong MY, Lee D, Hun Nam SH, Yoon HC, Kim HS (2005). Inhibition assay of biomolecules based on fluorescence resonance energy transfer (FRET) between quantum dots and gold nanoparticles. Journal of the american chemical society.

[57] Lovell JF, Chen J, Jarvi MT, Cao WG, Allen AD, Liu Y, Tidwell TT, Wilson BC, Zheng G (2009). FRET quenching of photosensitizer singlet oxygen generation. The journal of physical chemistry B.

[58] Rojas-Cervellera V, Raich L, Akola J, Rovira C (2017). The molecular mechanism of the ligand exchange reaction of an antibody against a glutathione-coated gold cluster. Nanoscale.

[59] Zhang Y, Clapp A (2011). Overview of stabilizing ligands for biocompatible quantum dot nanocrystals. Sensors (Basel).

[60] Frasco M, Chaniotakis N (2009). Semiconductor quantum dots in chemical sensors and biosensors. Sensors (Basel).

[61] Roy MD, Herzing AA, Lacerda SHDP, Becker ML (2008). Emission-tunable microwave synthesis of highly luminescent water soluble CdSe/ZnS quantum dots. Chemical communications.

[62] Vo N, Ngo HD, Vu DL, Duong AP, Lam QV (2015). Conjugation of E coli O157:H7 antibody to CdSe/ZnS quantum dots. Journal of nanomaterials.

[63] Javidparvar AA, Ramezanzadeh B, Ghasemi E (2016). Effects of surface morphology and treatment of iron oxide Javidparvar nanoparticles on the mechanical properties of an epoxy coating. Progress in organic coatings.

[64] Javidparvar AA, Ramezanzadeh B, Ghasemi E (2016). The effect of surface morphology and treatment of Fe3O4 nanoparticles on the corrosion resistance of epoxy coating. Journal of the Taiwan Institute of chemical engineers.

[65] Javidparvar AA, Naderi R, Ramezanzadeh B, Bahlakeh G (2019). Graphene oxide as a pH-sensitive carrier for targeted delivery of eco-friendly corrosion inhibitors in chloride solution: Experimental and theroretical investigations. Journal of industrial and engineering chemistry.

[66] Javidparvar AA, Naderi R, Ramezanzadeh B (2019). Designing a potent anti-corrosion system based on graphene oxide nanosheets non-covalently modified with cerium/benzimidazole for selective delivery of corrosion inhibitors on steel in NaCl media. Journal of molecular liquids.

[67] Jiménez-López J, Rodrigues S, Ribeiro D, Ortega-Barrales P, Medina AR, Santos JLM (2019). Exploiting the fluorescence resonance energy transfer (FRET) between CdTe quantum dots and Au nanoparticles for the determination of bioactive thiols. Spectrochimica acta part A: molecular and biomolecular spectroscopy.

[68] Nasirzadeh K, Nazarian S, Gheibi Hayat SM (2016). Inorganic nanomaterials: A brief overview of the applications and developments in sensing and drug delivery. Journal of applied biotechnology reports.

[69] Palmer T (1995). Kinetics of single-substrate enzyme catalysed reactions. Understanding enzymes.

[70] Cornish-Bowden A (2012). Fundamentals of enzyme kinetics (4th edition).

[71] Prieto-Simón B, Fàbregas E (2004). Comparative study of electron mediators used in the electrochemical oxidation of NADH. Biosensors and bioelectronics.

[72] Teymourian H, Salimi A, Hallaj R (2012). Low potential detection of NADH based on Fe3O4 nanoparticles/multiwalled carbon nanotubes composite: fabrication of integrated dehydrogenase-based lactate biosensor. Biosensors and bioelectronics.

[73] Tetianec L, Chaleckaja A, Kulys J, Janci R, Marcinkeviciene L, Meskiene R, Stankevciute J, Meskys R (2017). Characterization of methylated azopyridine as a potential electron transfer mediator for electroenzymatic systems. Process biochemistry.

[74] Ooi T, Shibata T, Sato R, Ohno H, Kinoshita S, Thuoc TL, /taguchi S (2007). An azoreductase, aerobic NADH-dependent flavoprotein discovered from Bacillus sp functional expression and enzymatic characterization. Applied microbiology and biotechnology.

[75] Qi J, Schlömann M, Tischler D (2016). Biochemical characterization of an azoreductase from rhodococcus opacus 1CP possessing methyl red degradation ability. Journal of molecular catalysis B enzymatic.

[76] Zhou M, Chen X, Xu Y, Qu J, Jiao L, Zhang H, Chen H, Chen X (2013). Sensitive determination of Sudan dyes in foodstuffs by Mn–ZnS quantum dots. Dyes and pigments.

[77] Zhang J, Na L, Jiang Y, Han D, Lou D, Jin L (2017). A fluorescence-quenching method for quantitative analysis of Ponceau 4R in beverage. Food chemistry.

[78] Mazlan SZ, Lee YH, Hanifah SA (2017). A new Laccase based biosensor for tartrazine. Sensors (Basel).

[79] Yin H, Zhou Y, Ai S, Chen Q, Zhu X, Liu X, Zhu L (2010). Sensitivity and selectivity determination of BPA in real water samples using PAMAM dendrimer and CoTe quantum dots modified glassy carbon electrode. Journal of hazardous materials.

[80] Dahan M, Lévi S, Luccardini C, Rostaing P, Riveau B, Triller A (2003). Diffusion dynamics of glycine receptors revealed by single-quantum dot tracking. Science.

[81] Härmä H, Soukka T, Lövgren T (2001). Europium nanoparticles and time-resolved fluorescence for ultrasensitive detection of prostate-specific antigen. Clinical chemistry.

[82] Larson DR, Zipfel WR, Williams RM, Clark SW, Bruchez MP, Wise F, Webb W (2003). Water-soluble quantum dots for multiphoton fluorescence imaging in vivo. Science.

[83] Qi L, Gao X (2008). Emerging application of quantum dots for drug delivery and therapy. Expert opinion on drug delivery.

[84] Wang HZ, Wang HY, Liang RQ, Ruan KC (2004). Detection of tumor marker CA125 in ovarian carcinoma using quantum dots. Acta biochimica et biophysica sinica.

[85] Derfus AM, Chen AA, Min DH, Ruoslahti E, Bhatia SN (2007). Targeted quantum dot conjugates for siRNA delivery. Bioconjugate chemistry.

[86] Franciscon E, Piubeli F, Fantinatti-Garboggini F, de Menezes CR, Silva IS, Cavaco-Paulo A, Grossman MJ, Durrant LR (2010). Polymerization study of the aromatic amines generated by the biodegradation of azo dyes using the laccase enzyme. Enzyme and microbial technology.

